# The effect of posterior cruciate ligament tibial avulsion fracture on functional outcomes in knees with concomitant ipsilateral lower limb fractures: a matched-cohort analysis

**DOI:** 10.1186/s12891-023-06529-0

**Published:** 2023-05-20

**Authors:** Hua-zhang Xiong, Hong-jie Yang, Lian-rong Du, Xiu-qi Liu, Lv Sun, Ying Jin, Li-ming Dong

**Affiliations:** 1 Department of Orthopedic Surgery, Affiliated Hospital of Zunyi Medical University, 149# Dalian Road, Zunyi, 563003 Guizhou Province People’s Republic of China; 2 Department of Radiology, Affiliated Hospital of Zunyi Medical University, Medical Imaging Center of Guizhou Province, Zunyi, Guizhou Province People’s Republic of China 563003

**Keywords:** Outcomes, Treatment, Posterior cruciate ligament, Tibial avulsion fracture, Combination, Ipsilateral lower limb fractures

## Abstract

**Background:**

At present, the optimal treatment for posterior cruciate ligament tibial avulsion fracture (PCLTAF) combined with concomitant ipsilateral lower limb fractures remains unclear. The present study aimed to assess the preliminary outcomes of treatment for PCLTAF with concomitant ipsilateral lower limb fractures by open reduction and internal fixation (ORIF).

**Materials and Methods:**

The medical records of patients who sustained PCLTAF with concomitant ipsilateral lower limb fractures between March 2015 and February 2019 and underwent treatment at a single institution were retrospectively reviewed. Imaging examinations performed at the time of injury were applied to identify concomitant ipsilateral lower limb fractures. We used 1:2 matching between patients with PCLTAF combined with concomitant ipsilateral lower limb fractures (combined group; *n* = 11) and those with isolated PCLTAF (isolated group; *n* = 22). Outcome data were collected, including the range of motion (ROM) and visual analogue scale (VAS), Tegner, Lysholm, and International Knee Documentation Committee (IKDC) scores. At the final follow-up, the clinical outcomes were compared between the combined and isolated groups and between patients who underwent early-stage surgery and those who underwent delayed treatment for PCLTAF.

**Results:**

Thirty-three patients (26 males, 7 females) were included in this study, with eleven patients having PCLTAF and concomitant ipsilateral lower limb fractures and a follow-up of 3.1 to 7.4 years (average, 4.8 years). Compared to patients in the isolated group, patients in the combined group demonstrated significantly worse Lysholm scores (85.7 ± 5.8 vs. 91.5 ± 3.9, *p* = 0.040), Tegner scores (4.4 ± 0.9 vs. 5.4 ± 0.8, *p* = 0.006), and IKDC scores (83.6 ± 9.3 vs. 90.5 ± 3.0, *p* = 0.008). Inferior outcomes were found in patients with delayed treatment.

**Conclusions:**

Inferior results were found in patients with concomitant ipsilateral lower limb fractures, while better outcomes were obtained in patients with PCLTAF through early-stage ORIF using the posteromedial approach. The present findings may help determine the prognoses of patients with PCLTAF combined with concomitant ipsilateral lower limb fractures treated through early-stage ORIF.

## Introduction

Posterior cruciate ligament tibial avulsion fracture (PCLTAF) is rare. The incidence of PCLTAF is still unknown due to its rarity [1–3]. PCLTAF is divided into three types according to the Meyers-McKeever classification [4]: in type I, there is no displacement of fracture fragments; in type II, there is a slightly displaced posterior margin with an intact anterior cortex acting as a hinge; and in type III, there is a fully displaced void of all bony contacts. If the PCLTAF is not displaced, conservative treatment may be suggested, while surgical reduction and fixation should be considered in patients with type II and III fractures.

PCLTAF with concomitant ipsilateral lower limb fractures is scarce, and a complex lower limb injury that can cause knee instability or, when associated with neurovascular injury, can threaten the involved limb. Although the actual incidence of a PCLTAF with concomitant ipsilateral lower limb fractures is unclear, these fractures are relatively uncommon. PCLTAF management seems to be easily neglected [5, 6], and inappropriate management can seriously impact knee function [5, 7, 8]. Currently, there have been few clinical studies on PCLTAF with concomitant ipsilateral lower limb fractures [6]. Joseph et al. [6] reported 3 patients with chronic PCLTAF combined with concomitant ipsilateral lower limb fractures in 2019; all patients underwent delayed open reduction and internal fixation (ORIF) of PCLTAF, and good outcomes were obtained. To the best of our knowledge, barring a few case reports, few studies have reported on PCLTAF with concomitant ipsilateral lower limb fractures. The relationship of PCLTAF results to whether concomitant ipsilateral lower limb fractures are present or absent has not yet been assessed. It is also unclear whether delayed treatment for PCLTAF with ipsilateral lower limb fractures affects functional results. Therefore, the primary objective of this study was to evaluate the effect of PCLTAF on functional outcomes in knees with concomitant ipsilateral lower limb fractures, and the secondary objective of this study was to compare the clinical outcomes between early-stage surgery and delayed treatment for PCLTAF. We hypothesized that PCLTAF with concomitant ipsilateral lower limb fractures has an adverse effect on knee outcomes and that treating PCLTAF in the early stage would lead to better outcomes than treating PCLTAF in the delayed stage.

## Material and methods

This was a retrospective study to assess the outcomes of ORIF for the treatment of PCLTAF with concomitant ipsilateral lower limb fractures. The study protocol was approved by the Ethics Committee of our institution (KLL-2022–772). All methods were performed in accordance with the Chinese Ethical Guidelines for Medical and Biological Research Involving Human Subjects, and each participant provided written informed consent.

### Participants

**Fig. 1:**
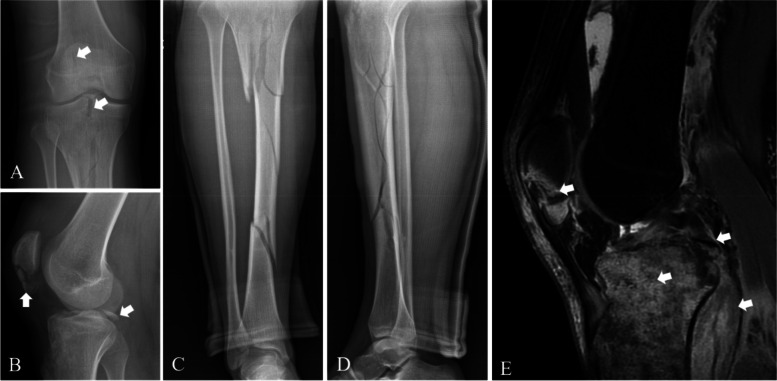
Preoperative right knee radiographs of an 18-year-old male: AP (**A**) and lateral (**B**) views showed patellar comminuted, proximal tibial comminuted and PCL tibial avulsion fractures. Preoperative tibial and fibular radiographs: AP (**C**) and lateral (**D**) views revealed multiple comminuted fractures. Preoperative sagittal fat-suppressed T2-weighted magnetic resonance imaging (MRI) of the right knee (**E**) demonstrated patellar and PCL tibial avulsion fractures and peri-knee soft tissue and tibial marrow oedema (white arrow)

**Fig. 2:**
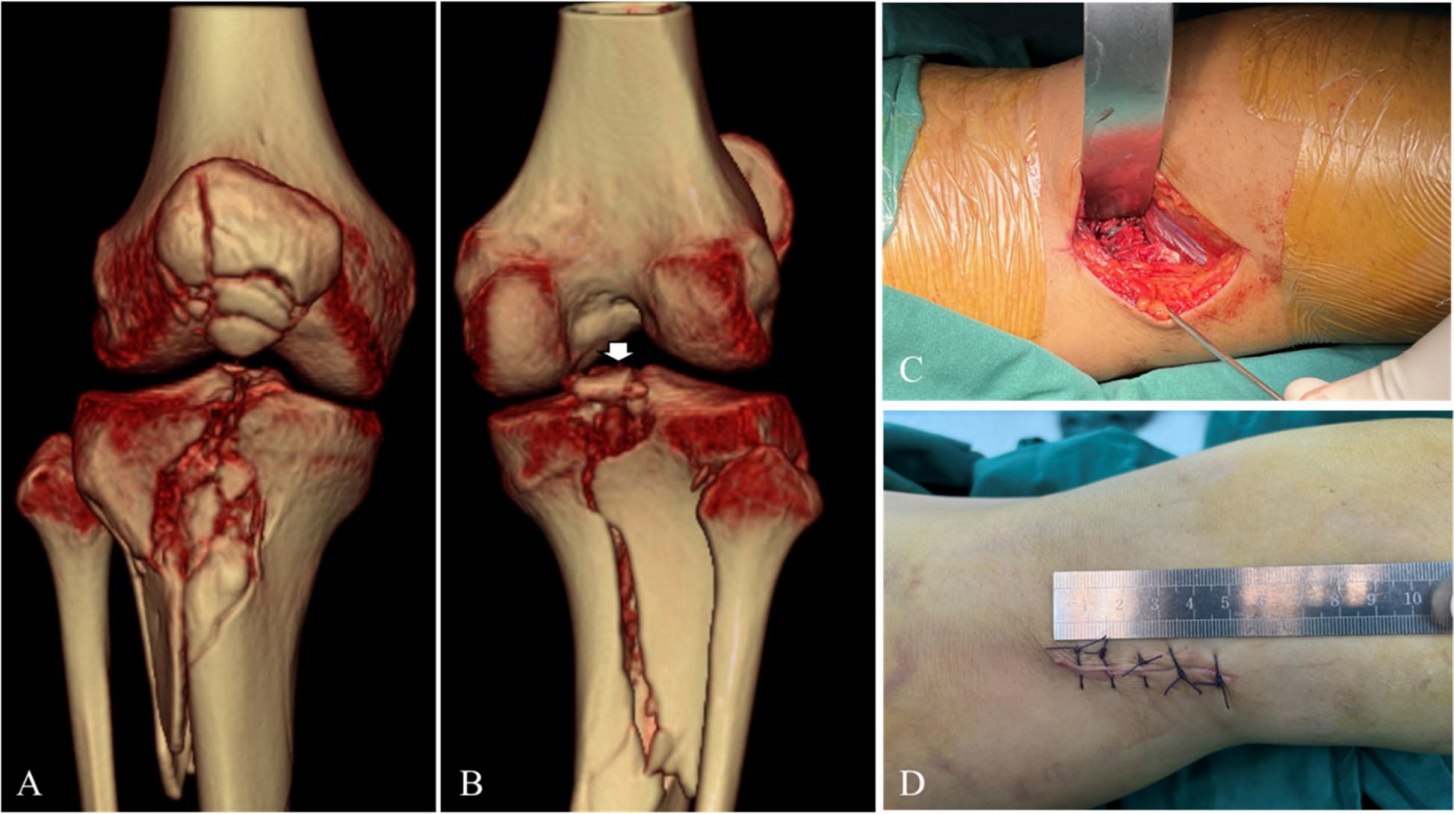
Preoperative knee three-dimensional (3D) computed tomography (CT): anterior (**A**) and posterior (**B**) views showed patellar comminuted, proximal tibial comminuted and PCL tibial avulsion fractures. The intraoperative incision pictures of the minimally invasive posteromedial approach showed the exposure of the operative site (**C**) with a 5 cm length incision (**D**)

All operations in this study were performed by one senior surgeon between March 2015 and February 2019. All patients who were diagnosed with PCLTAF combined with concomitant ipsilateral lower limb fractures were subsequently identified. A concomitant ipsilateral lower limb fracture was defined as a fracture of one or more of the following bones: the femur, patella, tibia, or fibula. We included participants in this study according to the literature’s methods [9, 10]. Inclusion criteria: patients with PCLTAF (type II and III fractures) [4] combined with concomitant ipsilateral lower limb fractures with a minimum follow-up of 3 years. Exclusion criteria: patients with single lower limb fracture(s) that were associated with contralateral limb fracture(s) or other injuries such as meniscal, ligamentous, and musculotendinous injuries requiring surgeries, who had any history of knee injury or surgery, and who were lost to follow-up; and patients with combined neurovascular injuries. The injuries of each patient were clearly diagnosed by preoperative X-ray, computed tomography (CT), and magnetic resonance imaging (MRI) (Figs. 1 and 2 A, B). The injuries of all patients are shown in Table 1.

**Table 1:** Detailed description of patients with PCLTAF combined with concomitant ipsilateral lower limb fractures (*n* = 11)

Patient No	Gender	Age(y)	Side	Cause	Combined fracture location	Combined fracture treatment	PCLTAF treatment	Time to surgery (Days)	Duration of surgery (Mins)
Case 1	Male	17	Right	Fall	Ti + Pa	ORIF	Early-stage ORIF	10	32
Case 2	Male	31	Right	Accident	Fe	ORIF	Early-stage ORIF	19	31
Case 3	Male	59	Left	Fall	Fe + Ti + Pa	ORIF	Early-stage ORIF	11	28
Case 4	Female	42	Right	Accident	Pa	ORIF	Early-stage ORIF	9	32
Case 5	Male	44	Left	Accident	Fe	ORIF	Early-stage ORIF	20	35
Case 6	Female	59	Right	Fall	Ti	ORIF	Early-stage ORIF	19	33
Case 7	Male	39	Left	Fall	Pa	ORIF	Early-stage ORIF	10	30
Case 8	Female	43	Right	Fall	Fe	ORIF	Early-tage ORIF	2	31
Case 9	Male	44	Left	Accident	Fe	ORIF	Delayed ORIF	372	46
Case 10	Male	45	Left	Accident	Ti + Fi	ORIF	Conservative	**492**^**a**^	-
Case 11	Male	55	Left	Accident	Fe + Ti	ORIF	Delayed ORIF	155	55

*Fe* femoral, *Fi* fibular, *ORIF* open reduction and internal fixation, *Pa* patellar, *PCLTAF* posterior cruciate ligament tibial avulsion fracture. *Ti* tibial

^a^Time to treatment

**Fig. 3:**
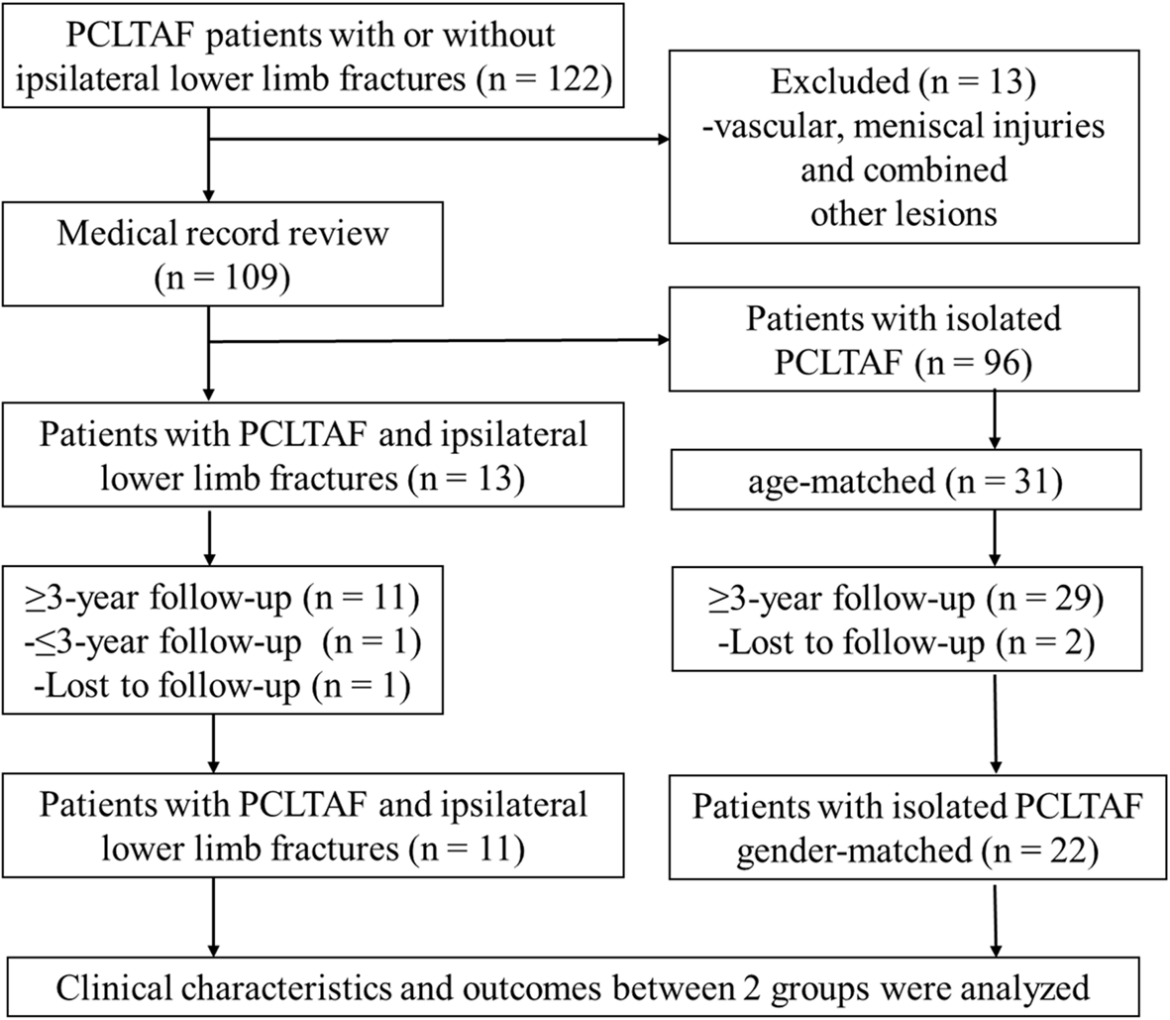
Flowchart of patient enrolment. PCLTAF, posterior cruciate ligament tibial avulsion fracture

For comparison of the combined group and isolated group, the patients with PCLTAF and concomitant ipsilateral lower limb fractures comprising the combined group were matched 1:2 with patients with isolated PCLTAF comprising the isolated group. After matching was performed, the final cohort was achieved. The following data were collected according to a standardized protocol: age at injury, sex, body mass index (BMI), and complications (Fig. 3). For the comparison of early-stage surgery and delayed treatment, the patients were divided on the basis of surgical timing. Early-stage surgery performed within the first 21 days after the injury was defined as being performed during the early phase, while delayed treatment that occurred more than 21 days after the injury was defined as being performed during the chronic phase [6].

### Surgical technique

The patients in the combined group first underwent ORIF of the concomitant ipsilateral lower limb fractures under general anaesthesia in the supine position on the operation table. The PCLTAFs were treated in the prone position under fluoroscopic guidance based on the approach described by Zhang et al. [11]. A thigh tourniquet was applied, and a straight incision was uniformly made overlying the medial head of the gastrocnemius. The incision originated from the transverse striation of the popliteal fossa skin and extended distally along the medial border of the gastrocnemius muscle with a length of 5–6 cm (Fig. 2 C, D). Blunt separation accessed the gap between the medial head of the gastrocnemius muscle and the semitendinosus muscle. During the surgery, the medial head of the gastrocnemius was pulled laterally to expose the posterior joint capsule using a deep retractor. After exposure, the joint capsule was then located and longitudinally incised. The PCL and fracture areas were completely exposed with the knee flexed 20 to 30°, and the fracture space was debrided and freshened. The avulsion fracture was reduced, and temporary fixation was performed through two 1.2 mm Kirschner wires. C-arm fluoroscopy confirmed that the anatomical reduction of the fracture was obtained, and the fracture was fixed using two 3.5 mm partially threaded cancellous lag screws with a washer. After fixation, a good position of the fracture was confirmed using C-arm fluoroscopy. Haemostasis was completely performed, and the operative site was repeatedly irrigated. The posterior capsule was closed to increase the stability of the knee joint, and the incision was sutured layer by layer. All individuals received prophylactic antibiotics and perioperative prophylactic anti-thrombotic management using low-molecular-weight heparin [12]. All patients were followed up at 1, 3, 6, and 12 months post-surgery and annually thereafter.

### Postoperative rehabilitation

**Fig. 4:**
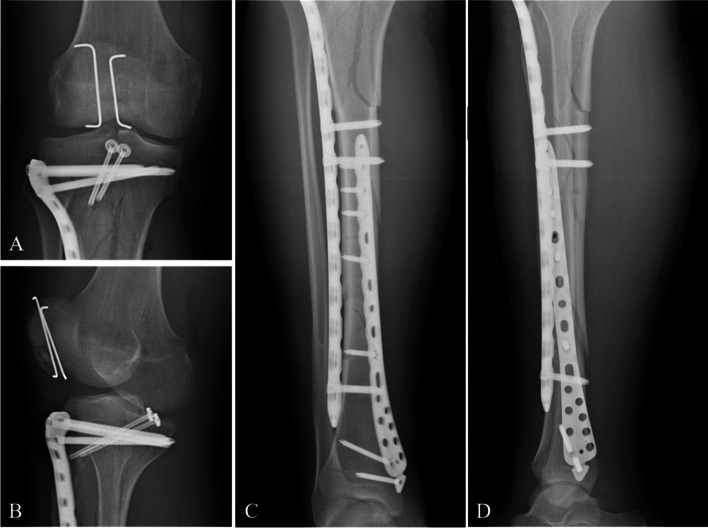
Postoperative radiographs: AP (**A**) and oblique (**B**) views of the right knee showed that the PCLTAF was treated through two partially threaded cancellous lag screws with washers, the patellar fractures were treated by ORIF using Kirschner wires, and the proximal tibial plateau fractures were managed by applying locked plates and screws. AP (**C**) and lateral (**D**) views of the tibia revealed that the tibial shaft fractures were managed using locked plates and screws

**Fig. 5:**
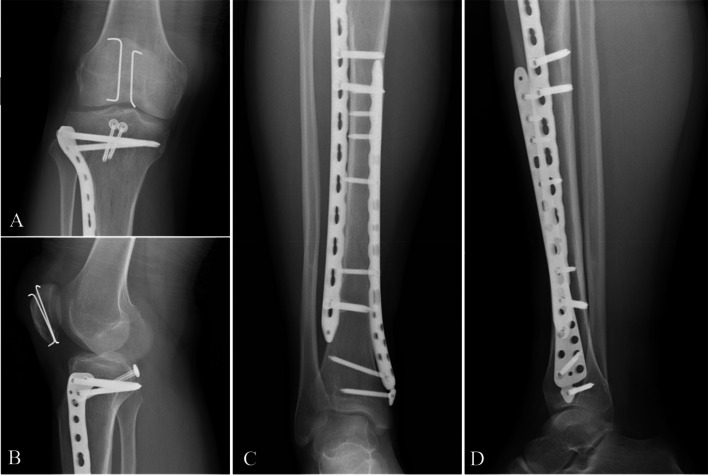
Postoperative radiographs at 3 months after surgery: AP (**A**) and lateral (**B**) views of the knee showed good healing of the PCL tibial insert, patella, and tibial plateau fractures. AP (**C**) and lateral (**D**) views of the tibia showed good healing in the tibial shaft fractures

Functional exercises were started as recommended by Zhang et al. [11]. The exercises strengthening the quadriceps and ankle flexion and extension muscles were isometric contractions starting one day after surgery. The involved knee was fixed at 5 to 10° of flexion using a locked hinged brace for 6 weeks, and after that, active flexion exercises were started for the involved knee, which was protected during walking with the hinged brace unlocked for 3 weeks. The range of motion (ROM) of the knee increased gradually, reaching that of the contralateral knee within 3 weeks. The patient was followed up to assess the healing of the fractures. X-ray was used at 2 days (Fig. 4) and 3 months (Fig. 5), and was then used if necessary at 6 months, 12 months, and thereafter annually after surgery. Radiographic examinations included anteroposterior (AP) and lateral radiographs at the knee. Patients were allowed to ambulate without the brace once radiographic evidence of union and clinical knee stability were obtained. The internal fixator was removed 12 months after the operation according to the patient's requirements.

### Data collection

The Lysholm score [13], International Knee Documentation Committee (IKDC) score [14], Tegner score [15], and ROM were used to evaluate knee function. Knee pain was evaluated by a visual analogue scale (VAS) [16]. The Lysholm score was used to evaluate subjective symptoms and grade, and a total score of ≥ 90 was defined as an excellent score, 84–90 as good, 65–83 as fair, and < 65 as poor. A series of self-administered questionnaires using the VAS and Lysholm, Tegner and IKDC scales were separately evaluated, and the ROM was measured through standardized goniometry methods by a senior author who was also blinded to surgical procedures at each follow-up. Complications were recorded.

### Statistical analyses

Before statistical analysis, data were independently reviewed and validated. Statistical tests were performed using SPSS software SPSS® version 22 (SPSS Inc., Chicago, Illinois) by a researcher who was also blinded to surgical procedures and data collection. All values are expressed as the mean values with standard deviations (SDs). The Kolmogorov–Smirnov test was performed on each continuous variable to determine normality. The Mann–Whitney U test was used for continuous variables. For categorical variables, chi-square or Fisher’s exact tests were used to compare the outcomes between the combined and isolated groups. The differences in values were considered statistically significant when the corresponding *p* value was < *0.05*.

## Results

### Demographic and clinical characteristics

After patients with PCLTAF and concomitant ipsilateral lower limb fractures (combined group, *n* = 11) were matched 1:2 with patients with isolated PCLTAF (isolated group, *n* = 22), 33 patients were included in this study (26 males, 7 females). Complete data were retrospectively reviewed and included for 33 patients. The data of patients with concomitant ipsilateral lower limb fractures and management are shown in Table 1. The mean age was 43.5 ± 12.2 years in the combined group and 42.9 ± 11.9 years in the isolated group, with an average follow-up of 5.5 ± 1.2 years and 4.9 ± 1.3 years, respectively. In the combined group, the mechanisms of injury differed and included a traffic accident (6 patients) and a fall injury (5 patients). In the isolated group, the mechanisms of injury were a fall in 17 patients, a traffic accident in 3 patients, and a twisting injury in 2 patients. Significant differences were not found in age, BMI, duration of follow-up, duration of surgery, or time to surgery between the two groups. The demographic data are shown in Table 2. Three months after ORIF, X-rays revealed healing of the fractures in all patients.

**Table 2:** Demographic characteristics of patients in the combined and isolated groups (*n* = 33)^b^

	Combined group (*n* = 11)	Isolated group (*n* = 22)	*p* value
Age (years)	43.5 ± 12.2	42.9 ± 11.9	0.904^a^
Gender			
Male	8 (72.7%)	16 (72.7%)	-
Female	3 (27.3%)	6 (27.3%)	-
Side			
Right	5 (45.5%)	10 (45.5%)	-
Left	6 (54.5%)	12 (54.5%)	-
BMI (kg/m^2^)	24.7 ± 1.2	24.6 ± 1.9	0.954^a^
Mechanism of injury			
Fall	5 (45.5%)	17 (77.3%)	-
Accident	6 (45.5%)	3 (13.6%)	-
Twist	0	2 (9.1%)	-
Time to treatment (days)	101.7 ± 168.4	47.3 ± 153.3	0.104^a^
Early-stage surgery	8 (72.7%)	18 (81.8%)	-
Delayed treatment	3 (27.3%)	4 (18.2%)	-
Duration of surgery	35.3 ± 8.5	32.8 ± 7.5	0.318^a^
Follow-up time (years)	5.5 ± 1.2	4.9 ± 1.3	0.187^a^
Complications			
Knee stiffness	1 (9.1%)	0	-
Knee arthritis	1 (9.1%)	0	-
ONFH	1 (9.1%)	0	-

^a^Independent‑sample Mann‑Whitney U test. *BMI* body mass index, *ONFH* osteonecrosis of the femoral head

^b^Continuous variables are expressed as the mean and the standard deviation. Categorical variables are presented as numbers with percentages in parentheses

### Functional outcomes

At the final follow-up, there were significant differences in the Lysholm scores (85.7 ± 5.8 vs. 91.5 ± 3.9, *p* = 0.040), Tegner scores (4.4 ± 0.9 vs. 5.4 ± 0.8, *p* = 0.006), and IKDC scores (83.6 ± 9.3 vs. 90.5 ± 3.0,* p* = 0.008) between the combined and isolated groups, while there were no significant differences in the ROM (118.6 ± 25.5 vs. 127.5 ± 5.7, *p* = 0.280) or VAS score (1.5 ± 1.3 vs. 0.7 ± 0.8, *p* = 0.108). The functional outcomes are shown in Table 3. The outcomes at the final follow-up in terms of the duration of surgery, Lysholm score, Tegner score, IKDC score, and VAS score were better for the patients with early-stage ORIF for PCLTAF than for the patients with delayed treatment. Detailed outcomes are shown in Table 4.

**Table 3:** Comparison of functional outcomes between the combined and isolated groups at the final follow-up^b^

Outcome measure	Combined group	Isolated group	*p* value
Lysholm score	85.7 ± 5.8	91.5 ± 3.9	**0.020**^**a**^
IKDC score	83.6 ± 9.3	90.5 ± 3.0	**0.003**^**a**^
Tegner Score	4.4 ± 0.9	5.4 ± 0.8	**0.004**^**a**^
VAS	1.5 ± 1.3	0.7 ± 0.8	0.108^a^
ROM	118.6 ± 25.5	127.5 ± 5.7	0.280^a^

*ROM* Range of motion, *VAS* Visual Analogue Score, *IKDC* International Knee Documentation Committee

^a^Independent‑sample Mann‑Whitney U test. Boldface indicates statistical significance

^b^Continuous variables are expressed as the mean and the standard deviation

**Table 4:** Comparison of the outcomes at the final follow-up between patients who underwent early-stage surgery and patients who underwent delayed treatment^a^

	All patients in the two groups			Isolated group				Combined group		
	Early-stage surgery(*n* = 26)	Delayed surgery(*n* = 7)	*p* value	Early-stage surgery(*n* = 18)	Delayed surgery(*n* = 4)	*p* value	Early-stage surgery(*n* = 8)		Delayed surgery(*n* = 3)	*p* value
Lysholm score	**91.4 ± 3.4**	**82.6 ± 5.4**	**0.000**	**92.9 ± 2.1**	**85.0 ± 3.5**	**0.000**	**88.1 ± 3.6**		**79.3 ± 6.4**	**0.016**
IKDC score	89.7 ± 3.6	82.5 ± 11.6	0.151	90.5 ± 3.1	90.3 ± 3.0	0.915	**88.0 ± 4.0**		**72.0 ± 10.1**	**0.003**
Tegner Score	**5.4 ± 0.7**	**3.9 ± 0.7**	**0.000**	**5.7 ± 0.5**	**4.0 ± 0.8**	**0.000**	4.8 ± 0.7		3.7 ± 0.6	0.054
VAS	0.8 ± 0.8	1.7 ± 1.5	0.151	0.7 ± 0.8	0.75 ± 1.0	0.95	**0.88 ± 0.8**		**3.0 ± 1.0**	**0.044**
ROM (°)	128.5 ± 3.9	110.0 ± 30.3	0.158	128.1 ± 4.2	125.0 ± 10.8	0.614	129.4 ± 3.2		90.1 ± 30.1	0.223
Duration of surgery	**30.3 ± 3.2**	**47.8 ± 4.5**^b^	**0.000**	**29.7 ± 3.5**	**46.5 ± 3.7**	**0.000**	31.5 ± 2.1		50.5 ± 6.4^b^	0.140

*IKDC* International Knee Documentation Committee, *ROM* Range of motion, VAS: Visual Analogue Score

^a^Continuous variables are expressed as the mean and the standard deviation. The variables a were tested using the independent‑samples Mann‑Whitney U test. Boldface indicates statistical significance

^b^One patient did not undergo delayed surgery for PCLTAF

In the combined and isolated groups, the Lysholm score of the involved knee at the final follow-up was excellent in 4 (36.3%) and 18 (81.8%) patients, good in 5 (45.5%) and 3 (13.6%) patients and fair in 2 (18.1%) and 1 (4.5%) patient, respectively. Overall, in the combined group, early-stage ORIF was performed in 8 patients within the first 3 weeks. Three patients had a delay in diagnosis (22, 23, and 70 weeks), and ORIF procedures were performed in 2 patients. In another patient, the concomitant tibial plateau fracture (Schatzker II) and fibular shaft fracture were managed with ORIF, and PCLTAF was neglected. He was diagnosed with a left chronic PCLTAF with traumatic knee arthritis (Kellgren-Lawrence II-III) [17], knee stiffness, and irreducible posterior knee dislocation in our department one year after injury. He was recommended to undergo delayed total knee arthroplasty (TKA). At the last follow-up, he continued to choose conservative treatment with a limited ROM (5°-50°) and a VAS score of 4 and still did not undergo TKA.

Complications were reported by 2 patients. Knee stiffness with a ROM of 5°-50° and traumatic arthritis and osteonecrosis of the femoral head occurred in patient 10 and patient 9 in the combined group, respectively. There were no other postoperative complications in any patient, including incision infection or neurovascular injuries.

## Discussion

The most important finding of the present study was that the functional outcomes were worse for the patients in the combined group than for those in the isolated group at midterm follow-up. Better results were found in patients who underwent early-stage surgery. Early-stage surgery might be an ideal method for the treatment of PCLTAF with ipsilateral lower limb fractures.

PCLTAF can be treated by either open or arthroscopic methods [18, 19]. An arthroscopically assisted method might be beneficial for reducing operative traumas. However, the arthroscopic method requires a longer duration of surgery and has higher medical costs than the open procedure due to the necessary expertise and equipment [11, 19, 20], and it is unsuitable in less developed areas. Furthermore, arthroscopy-assisted internal fixation in the treatment of PCLTAFs has difficulty in achieving anatomical reduction [21, 22] and cannot easily release the contractured PCL and completely clean and freshen the fracture surface in patients with delayed treatment.

Several studies [18, 19, 23, 24] have reported that PCLTAF can be managed with satisfactory outcomes. However, none of the previous studies compared patients with PCLTAF and concomitant ipsilateral lower limb fractures to those with isolated PCLTAF. The present study is unique from previous studies [5, 18, 19, 23–26] of PCLTAF in that it reveals an association between the presence of concomitant ipsilateral lower limb fractures in patients with PCLTAF and worsened functional outcomes using Lysholm, Tegner, IKDC, and VAS scores and ROM. Significant differences between PCLTAF patients with and without ipsilateral lower limb fractures were found in the Lysholm, Tegner, and IKDC scores but not in the VAS score or ROM. The VAS score and ROM measures demonstrated worsened outcomes in PCLTAF patients with concomitant ipsilateral lower limb fractures that were close to but did not achieve significant differences. These differences might be explained by the following reasons: this could be a small sample size and underpowered study; it may be possible that muscle atrophy and the reduced proprioception can reduce patients’ pain and self-perceived function without affecting their pain or ROM; and lower limb fractures at different sites may also have influenced the results of this study. These findings may explain why a significant difference was found in the Lysholm, Tegner, and IKDC scores but not in the VAS score or ROM. The presence of concomitant ipsilateral lower limb fractures may be indicative of a higher energy trauma with severe and extensive injuries to the soft tissue and bone beyond the isolated PCLTAF. A greater area of tissue injury may lead to a more severe inflammatory response and put patients at risk for lower functional scores, which portend a worse prognosis.

This study assessed the effect of early-stage surgery with ORIF on the functional results in patients with PCLTAF and concomitant ipsilateral lower limb fractures. Several authors [5, 25, 26] have reported inferior outcomes with delayed treatment for isolated PCLTAF. In our study, the outcomes (including duration of surgery, Lysholm score, Tegner score, IKDC score, and VAS score) for early-stage ORIF of PCLTAF with and without ipsilateral lower limb fractures were found to be superior to those of delayed treatment, although some differences did not reach statistical significance. The inferior results of knee scores and ROM in patients with delayed treatment may be related to complications, such as traumatic arthritis and secondary knee dislocation, not necessarily due to PCLTAF itself (case 10). The treatment of PCLTAF is neglected and not scheduled; prolonged neglect may lead to long-term muscle atrophy and weakness, and it also reasonably decreases the functional score. Hence, the importance of initial diagnosis and treatment should be emphasized. ORIF may optimally be performed at the earliest stage. At our department, PCLTAFs with ipsilateral lower limb fractures were uniformly managed in the early stage with ORIF. In this study, at the latest follow-up, 9 of these 11 patients with early-stage fixation had excellent-good functional results. Early-stage ORIF can obtain acceptable outcomes in patients with PCLTAF and concomitant ipsilateral lower limb fractures.

There are several limitations to this study. First, this cohort analysis had a small sample size due to the low incidence of this injury. Second, there was inevitable heterogeneity among patients regarding the different locations of the concomitant ipsilateral limb fractures and operations. We collected all possible data with respect to PCLTAF associated with concomitant ipsilateral femoral, patellar, or tibial fractures close to the knee and performed analysis to decrease the impact of potential interfering factors. Finally, this study had a retrospective design, increasing the selection bias risk. However, this study shows that the early diagnosis and appropriate treatment of patients with PCLTAF combined with ipsilateral lower limb fracture(s) can achieve better knee functional outcomes.

## Conclusion

Inferior results were found in patients with concomitant ipsilateral lower limb fractures, while better outcomes were obtained in patients with PCLTAF through early-stage ORIF using the posteromedial approach. The present findings may help determine the prognoses of patients with PCLTAF combined with concomitant ipsilateral lower limb fractures treated through early-stage ORIF.

## Data Availability

The datasets generated and/or analyzed during the current study are not publicly because we will enlarge the sample size and extend the follow-up time to further explore the relationship between posterior cruciate ligament tibial avulsion fracture and clinical outcome, but these are available from the corresponding author on reasonable request.

## Abbreviations

PCLTAF: Posterior cruciate ligament tibial avulsion fracture
ORIF: Open reduction and internal fixation
ROM: Range of motion
VAS: Visual analogue scale
IKDC: International Knee Documentation Committee
